# Monoclonal Antibodies Recognizing the Non-Tandem Repeat Regions of the Human Mucin MUC4 in Pancreatic Cancer

**DOI:** 10.1371/journal.pone.0023344

**Published:** 2011-08-23

**Authors:** Maneesh Jain, Ganesh Venkatraman, Nicolas Moniaux, Sukhwinder Kaur, Sushil Kumar, Subhankar Chakraborty, Grish C. Varshney, Surinder K. Batra

**Affiliations:** 1 Department of Biochemistry and Molecular Biology, University of Nebraska Medical Center, Omaha, Nebraska, United States of America; 2 Eppley Institute for Research in Cancer and Allied Diseases, University of Nebraska Medical Center, Omaha, Nebraska, United States of America; 3 Institute of Microbial Technology, Chandigarh, India; Roswell Park Cancer Institute, United States of America

## Abstract

The MUC4 mucin is a high molecular weight, membrane-bound, and highly glycosylated protein. It is a multi-domain protein that is putatively cleaved into a large mucin-like subunit (MUC4α) and a C-terminal growth-factor like subunit (MUC4β). MUC4 plays critical roles in physiological and pathological conditions and is aberrantly overexpressed in several cancers, including those of the pancreas, cervix, breast and lung. It is also a potential biomarker for the diagnosis, prognosis and progression of several malignancies. Further, MUC4 plays diverse functional roles in cancer initiation and progression as evident from its involvement in oncogenic transformation, proliferation, inhibition of apoptosis, motility and invasion, and resistance to chemotherapy in human cancer cells. We have previously generated a monoclonal antibody 8G7, which is directed against the TR region of MUC4, and has been extensively used to study the expression of MUC4 in several malignancies. Here, we describe the generation of anti-MUC4 antibodies directed against the non-TR regions of MUC4. Recombinant glutathione-S-transferase (GST)-fused MUC4α fragments, both upstream (MUC4α-N-Ter) and downstream (MUC4α-C-Ter) of the TR domain, were used as immunogens to immunize BALB/c mice. Following cell fusion, hybridomas were screened using the aforementioned recombinant proteins ad lysates from human pancreatic cell lines. Three anti MUC4α-N-Ter and one anti-MUC4α-C-Ter antibodies were characterized by several inmmunoassays including enzyme-linked immunosorbent assay (ELISA), immunoblotting, immunofluorescene, flow cytometry and immunoprecipitation using MUC4 expressing human pancreatic cancer cell lines. The antibodies also reacted with the MUC4 in human pancreatic tumor sections in immunohistochemical analysis. The new domain-specific anti-MUC4 antibodies will serve as important reagents to study the structure-function relationship of MUC4 domains and for the development of MUC4-based diagnostics and therapeutics.

## Introduction

Human MUC4 is a highly glycosylated membrane-associated mucin, consisting of a large 850-kD mucin-like subunit MUC4α, and a membrane-bound 80 kD growth factor-like subunit MUC4β [1], [2]. MUC4α contains a central tandem repeat (TR) domain containing variable numbers of 16 amino-acid residue motifs that could be repeated up to 400 times per molecule. The TR domain is flanked by a C-terminal cysteine rich domain and an N-terminal domain which contains three repeats of 123 amino acid residues [1]. MUC4β contains a cysteine rich domain, a domain rich in N-glycosylation sites and three EGF-like domains [1]. MUC4 is considered to be a human homologue of rat sialo-mucin complex (SMC, rat Muc4) because of similarities in structural organization [1], [3], [4]. SMC is a heterodimeric glycoprotein composed of an O-glycosylated mucin subunit, ascites sialoglycoprotein (ASGP-1), tightly bound to a N-glycosylated transmembrane subunit, ASGP-2, which contains two epidermal growth factor-like domains in its extracellular part [3], [4].

MUC4 is expressed in various epithelial tissues, including the epithelia of fetal lungs and the adult respiratory tract from the trachea to the collecting ducts lung trachea [5], colon [6], endocervix [7], conjunctiva [8], cornea [9], salivary glands [10], middle ear and eustachian tube [11]. In recent studies, a progressive increase in MUC4 expression has been observed in pancreatic intraepithelial neoplastic lesions, indicating its role in disease development [12]. Previous studies from our laboratory have shown that inhibition of MUC4 expression using anti-sense or short-interfering RNA (siRNA) oligonucleotides specific to MUC4 results in a decreased tumorigenicity and dissemination of cancer cells [13]. Further, our recent studies have demonstrated that MUC4 results in oncogenic transformation of mouse fibroblasts [14], contributes to the drug-resistance of pancreatic cancer cells by activating anti-apoptotic pathways [15], and is involved in the epithelial-to-mesenchymal transition in ovarian cancer cells [16]. These studies from our laboratory and other groups indicate the potential importance of this mucin in various aspects of tumor biology.

We have previously generated a panel of monoclonal antibodies directed against the TR region of MUC4 [17]. One of the anti-MUC4 TR antibodies, 8G7, has served as a valuable reagent to study the expression of the MUC4 mucin in various tissues and unravel its involvement in various malignancies including, pancreatic [12], [18], gastric [19], cervical [20], ovarian cancers [21], extra hepatic bile duct carcinoma [22], colangiocarcinoma [23], and cutaneous squamous cell carcinoma. However, MUC4 contains many structural and functional domains both upstream and downstream of the TR region [1], [2], and many spliced forms of MUC4 are completely devoid of TR region [24], [25]. Further, the TR region is heavily O-glycosylated. Given the alteration in glycosylation status of solid tumors, it is possible that reactivity to the antibody can be obscured in certain malignancies. Thus, the structural complexity of MUC4, the existence of numerous splice variants and glycoforms, and heavy O-glycosylation in the TR domain warranted the generation of additional antibodies to fully understand the structure-function relationship of various MUC4 domains under physiological and pathological conditions.

Here, we report the generation and characterization of a novel anti-MUC4 MAbs that recognize the regions of MUC4α both upstream and downstream of the TR domain. Purified recombinant MUC4 fragments, fused in frame with GST, were used as immunogens and positive clones were selected based on their reactivity in ELISA. Selected clones were characterized by their reactivity toward MUC4 in immunoblotting, immunoprecipitation, immunofluorescence and flow cytometry using pancreatic cancer cells. The non-TR anti-MUC4 MAbs developed in this study may be promising reagents for the development of assays for quantification of MUC4 in tissues and biological fluids, to study the functional role of MUC4 in various diseases and potentially for immunotherapy.

## Results

**Figure 1:**
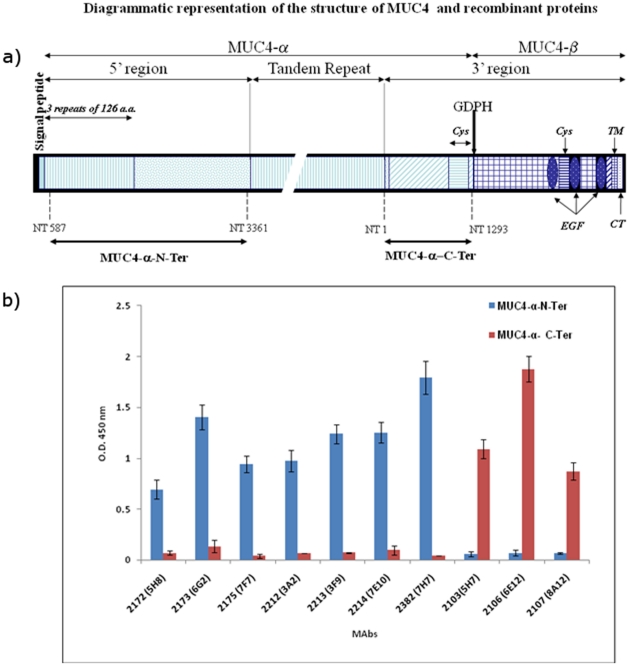
Schematic structure of the recombinant MUC4 domains and reactivity of various anti-MUC4 antibodies. **a**) Schematic structure of MUC4 and recombinant proteins used in the study. MUC4 is putatively cleaved at the GDPH site to generate an N-terminal mucin-type subunit MUC4-α and a C-terminal growth factor-type subunit MUC4-β. Important domains of MUC4 are marked. Recombinant domains of MUC4- α corresponding to the fragments upstream and downstream of the tandem-repeat (TR) domain were cloned and expressed as described in Materials and Methods and termed MUC4-α-N-ter and MUC4-α-C-Ter, respectively. The nucleotide numbers corresponding to the boundaries of the recombinant domains are marked and are described in Moniaux et al. and Choudhury et al (Ref 1 and 24, respectively) according to the original numbering. Cys-cystein-rich domain EGF-epidermal growth factor-like domain; TM-transmembrane domain; CT-cytoplasmic tail. **b**) ELISA showing the reactivity of anti-MUC4 MAbs to recombinant immunogens. The indicated MAbs were incubated with the 2.5 µg/ml of GST-tagged N-terminal and tandem repeat recombinant domains of MUC4. The specificities were also tested against the MUC4 TR peptide, GST and a non-specific control protein bovine serum albumin and the antibodies exhibited negative reactivity against these antigens. The assay also included a non-specific isotype matched control K2G6.

The schematic structure of MUC4 and the recombinant domains are indicated in **Figure 1a**. Following cell fusion, culture supernatants from stable hybridomas were screened and the positive hybridomas exhibiting high reactivity with the recombinant protein and negative reactivity with GST were cloned by three rounds of limiting dilution. Seven stable clones reactive with MUC4α-N-Ter and three clones reactive with MUC4α-C-Ter were obtained (**Table 1** and **Figure 1b**). MAbs 2172, 2173, 2175, 2212, 2213, 2214 and 2382 exhibited specific reactivity toward MUC4α-N-Ter, while MAbs 2103, 2106 and 2107 were specific to MUC4α-C-Ter. Further, none of the selected antibodies showed any reactivity toward purified MUC4 TR peptide, BSA or GST (data not shown). Similarly, previously generated anti-MUC4 TR antibody 8G7 or anti-KLH antibody K2G6 showed no reactivity toward the recombinant MUC4 domains.

**Table 1:** Nomenclature, isotype and origin of Non-TR anti MUC4 MAbs.

Clone ID	Immunogen	Isotype
2172 (5H8)	MUC4α-N-Ter	IgG2b, k
2173 (6G2)	MUC4α-N-Ter	IgG1, k
2175 (7F7)	MUC4α-N-Ter	IgG1, k
2212 (3A2)	MUC4α-N-Ter	IgG1, k
2213 (3F9)	MUC4α-N-Ter	IgG1, k
2214 (7E10)	MUC4α-N-Ter	IgG1, k
2382 (7H7)	MUC4α-N-Ter	IgG1, k
2103(5H7)	MUC4α-C-Ter	IgM, k
2106 (6E12)	MUC4α-C-Ter	IgG1, k
2107 (8A12)	MUC4α-C-Ter	IgG1, k
8G7	MUC4-TR peptide	IgG1, k
K2G6	KLH	IgG1, k

The generation of control MAbs 8G7 and K2G6 has been described in Moniaux et al (Ref 17).

**Figure 2:**
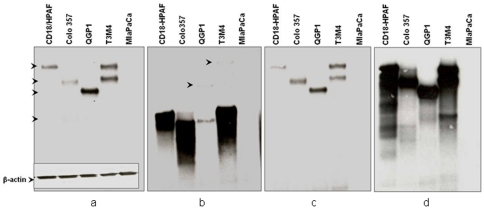
Comparative immunoblot analysis for MUC4 expression in various pancreatic cancer cell lines using various antibodies. A total of 20 µg of protein from cell extracts was resolved by electrophoresis on a 2% SDS-agarose gel, transferred to PVDF membrane, and incubated with 2 µg/ml of MAbs 2175 (a), 2214(b), 2382 (c) or 1 µg/ml of anti-MUC4 TR Mab 8G7(d). The membrane was then probed with horseradish peroxidase-labeled goat anti-mouse immunoglobulin. The signal was detected using an ECL reagent kit. The position of the detected bands is indicated by arrows. For loading control, immunoblot for the detection of β-actin (inset a) was done on lysates of respective cells resolved on 10% SDS-PAGE.

The antibodies were further tested for their ability to specifically recognize the MUC4 protein in the lysates of MUC4 expressing pancreatic cancer cell lines by immunoblotting. Of the seven MUC4α-N-Ter-specific antibodies only MAbs 2214, 2175 and 2382 recognized the MUC4 protein in the cell lysates (**Figure 2**). MAbs 2215 and 2382 recognized high molecular weight protein bands in the lysates of the MUC4 positive cells (HPAF/CD18, Colo357, QGP1 and T3M4) (Fig. 2a and 2c) and the reactivity pattern was similar to that of anti-TR MAb 8G7 (Fig. 2d). Each of the MUC4 positive cell lines exhibited a characteristically distinct band size which is consistent with our previous reports of VNTR polymorphisms in MUC4 with HPAF/CD18, Colo357 and QGP1 showing a single band and T3M4 expressing two bands (allelic VNTR polymorphism). Unlike MAbs 2175, 2382 and 8G7, MAb 2214 reacted predominantly with the low molecular weight form of MUC4 but with the band pattern corresponding to the VNTR polymorphism (Figure 2b). Mab 2214 also showed very weak reactivity with the high molecular band corresponding to those recognized by other antibodies in QGP1 and T3M4 lysates. Immunoblot analysis of β-actin in the SDS-PAGE resolved lysates indicated equal protein loading (**Figure 2** **, inset**). No reactivity was observed with any antibody with the lysate of the MUC4 negative cell line MiaPaCa. None of the anti-MUC4α-C-Ter antibodies reacted with MUC4 in the cell lysates in immunoblotting (data not shown).

**Figure 3:**
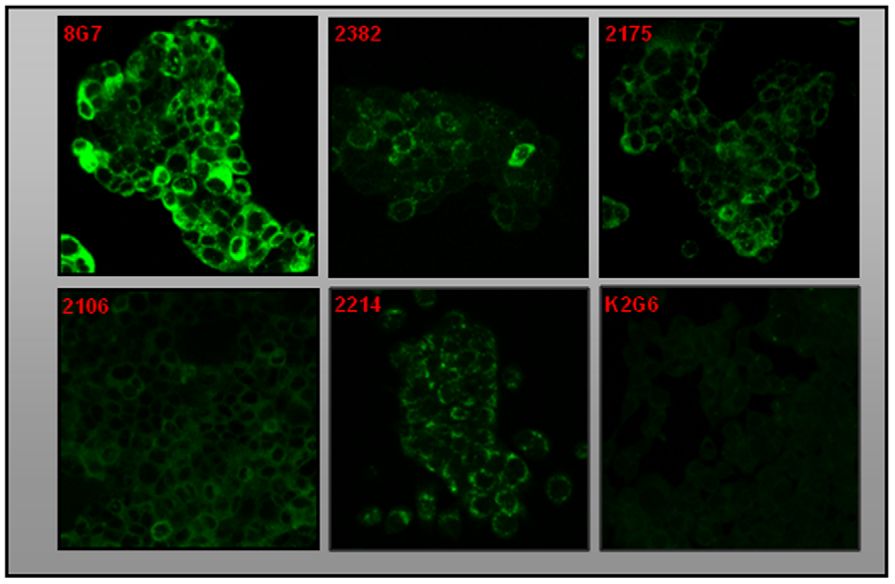
Immunofluorescence of MUC4 in CD18/HPAF cells with various anti-MUC4 MAbs. Cells were grown at low density on sterilized cover-slides, fixed in ice-cold methanol at −20°C and were incubated with 10 µg/ml non-TR MAbs of 2214, 2175, 2382 and 2106, or 2 µg/ml of anti-MUC4 TR MAb 8G7 (Control) and detected using FITC conjugated secondary antibody. Anti-KLH antibody K2G6 was used as an isotype control. Cells were mounted on glass slides using anti-fade Vectashield mounting medium and observed under a ZEISS confocal laser scanning microscope (magnification, ×630).

**Figure 4:**
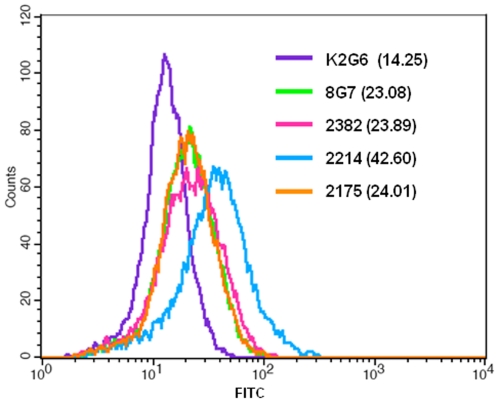
Cell-surface binding analysis of anti-MUC4 antibodies. Cells were harvested non-enzymatically, fixed with paraformaldehyde and incubated with the indicated antibodies. Following incubation with secondary antibody, cells were analyzed using BD FACSCalibur. The mean fluorescence intensities (MFI) values obtained with each antibody is indicated in parantheses.

The ability of antibodies to recognize MUC4 in the intact cells was studied by immunofluorescence and flow cytometry. In the methanol fixed and permeabilized assay HPAF/CD18 cells all the selected MAbs exhibited specific staining for MUC4; no staining was observed with the control anti-KLH antibody K2G6 (**Figure 3**). MAb 2214 showed a both membrane and perinuclear staining, while MAbs 2175, 2382 and 2106 showed cytoplasmic and membrane staining. The anti-TR MAb 8G7 showed strongest reactivity due to the repetitive nature of the epitopes. Further, none of the antibodies showed any reactivity with MUC4 negative pancreatic cancer cell lines MiaPaCa or Panc1 (data not shown). For cell surface staining, parformaldehyde-fixed (unpermmeabilized) cells were used and the binding of the antibodies was analyzed by flow cytometry. MAb 2214 exhibited the strongest reactivity with the cell surface in paraformaldehyde-fixed cells, while the surface reactivity of MAbs 2175 and 2382 was weak and the mean fluorescence intensity (MFI) values were comparable to the values obtained with MAb 8G7 (**Figure 4**).

**Figure 5:**
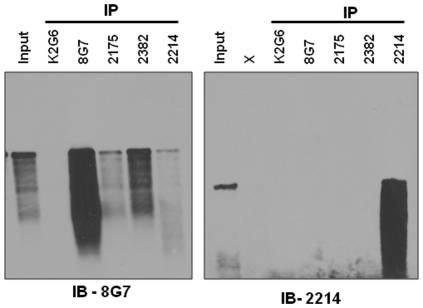
Immunoprecipitation of MUC4 using various MAbs to MUC4. Protein lysates from the MUC4-expressing CD18/HPAF cells were immunoprecipitated using 5 µg/ml of 8G7 (Tandem repeat MAb), 2382, 2214 and 2175 (Non-tandem repeat MAbs) and K2G6 (Isotype matched control MAb) and were immunoblotted using MAbs 8G7 and 2214 as described in Materials and Methods.

The domain-specific anti-MUC4 antibodies were also tested for their ability to immunoprecipitate MUC4 using the HPAF/CD18 lysate. MAbs 2382 2175, and 2214 immunoprecipitated full-length MUC4 from the total cell lysates, which was visualized when the processed samples were resolved on SAS-agarose gel and immunoblotted with anti-MUC4-TR MAb 8G7 (**Figure 5**). The immunoprecipitated samples from various antibodies were also immunoblotted with MAb 2214 due to its predominant reactivity with a lower molecular weight form of MUC4. When probed with MAb 8G7, the highest amount of MUC4 was immunoprecipitated with 8G7, while MAb 2382 also resulted in considerable enrichment of the 8G7 reactive protein bands. MAbs 2175 and 2214 also immunoprecipitated the full-length 8G7 reactive band but the enrichment was not as strong as observed with MAbs 8G7 and 2382. Anti-C-terminal MAb 2106 and negative control anti-KLH antibody K2G6 did not pull down any 8G7 reactive protein band. However, none of the tested antibodies except 2214, immunopecipitated the MAb 2214-reactive low molecular weight form of MUC4.

**Figure 6:**
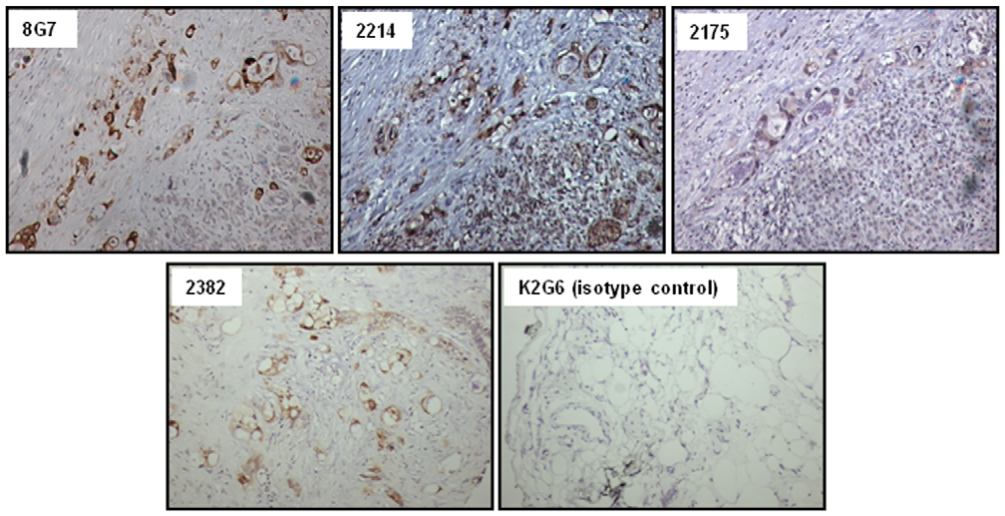
Immunoperoxidase staining for MUC4 in pancreatic cancer tissues using non-TR MAbs. Paraffin sections were incubated with the indicated test and control antibodies and binding was detected using VECTOR Universal staining Kit. MAb 8G7 was used at a concentration of 2 µg/ml, while all other antibodies were used at a concentration of 10 µg/ml.

The ability of antibodies to detect MUC4 in tumor tissues was tested by immunohistochemical analyses performed on pancreatic cancer tissues. MAbs 2214, 2175 and 2382 showed positive staining in the tumor tissue that was determined to be MUC4 positive based on its reactivity with anti-TR MAb 8G7 (**Figure 6**). The pattern of staining with the new antibodies was similar to that observed with 8G7 showing diffuse staining in both the membrane and the cytoplasm of the tumor cells. No staining was observed with Mab 2106 or the non-specific isotype matched control MAb K2G6.

## Discussion

MUC4 is a large glycoprotein involved in physiology and implicated in various disease states. Of particular importance is its role in pancreatic cancer development and progression [2], [26], [27]. A number of recent studies have established the role of the transmembrane mucin MUC4 in the pathogenesis of several malignancies. MUC4 consists of two domains, namely MUC4α which has the tandem repeat region and MUC4β which has the trans-membrane region and also possesses growth factor like domains [1], [2]. Due to the polymorphism in the number of tandem repeats [28] and the existence of various splice forms completely devoid of the TR domain [25], the antibodies recognizing the non-tandem repeat regions of the protein that could provide useful information about its function, possible interacting partners and more importantly can be used in quantitative assays.

Three of the antibodies raised against the region upstream of the central TR domain 2214, 2175 and 2382, and one of the antibodies generated against the downstream of the TR domain, 2106 showed strong reactivity against the respective recombinant domains in ELISA. None of the antibodies recognize the non-specific recombinant domains, GST or synthetic TR peptides. These antibodies can potentially serve as useful reagents for the development of MUC4 bioassays and can complement the existing anti-MUC4 TR antibody or other antibodies reactive against the carbohydrate epitopes present on mucins (DUPAN2, CA 19.9, TAG 72). Growing evidence suggests that the MUC4 mucin, due to its overexpression in several malignancies, is a potential marker for diagnosis [27], particularly for the lethal pancreatic cancer where its association with the early neoplastic lesions has been established [29]. Another recent study has shown MUC4 to be a novel prognostic factor of extra-hepatic bile duct carcinoma [22]. MUC4 expression was correlated with poor prognosis in small-sized lung adenocarcinoma [30]. All of these studies have shown that MUC4 could be a key player in tumorigenesis; however, all of these studies have analyzed MUC4 in tissue samples, which could be limited by sampling errors, due to the heterogeneous expression of tumor antigens. Hence, it would be logical to develop quantitative assays for MUC4 in biological fluids, which will be non-invasive, cost effective and easily automated. Due to the variable size of the tandem repeat region, the antibody recognizing the tandem repeat region could not be used for quantitative purposes. The domain specific antibodies can potentially aid in developing *in vitro* diagnostic assays to quantitate MUC4 in serum and other biological fluids.

All the antibodies reactive with the region upstream of the MUC4 TR domain were able to recognize MUC4 in the cell lysates of MUC4-expressing pancreatic cancer cells. MAbs 2175 and 2382 recognized the full-length MUC4 with a high molecular weight, with a band size similar to that recognized by anti-TR MAb 8G7. The difference in signal strength of the non-TR and TR antibodies could be attributed to the number of epitopes available for the MAb to bind, since 8G7 recognizes the tandem repeat region, which is represented multiple times in each molecule, whereas the epitopes recognized by 2175 and 2382 are represented only once per molecule. In contrast, Mab 2214 exhibited strong recognition of a protein band of smaller size than those recognized by MAbs 8G7, 2175 and 2382. Despite their lower molecular size, these bands mirrored the allelic variation exhibited by the full-length MUC4 for the respective cell lines, suggesting that Mab 2214 possibly reacts with an immature or underglycosylated form of MUC4. Very faint bands corresponding to the high molecular weight mature protein were still detected in QGP1 and T3M4. The stronger signal strength of Mab 2214 with the lower bands could be due to the abundance of an immature MUC4 protein in the cancer cells. In cancer cells it is well established that, due to aberrant and inefficient glycosylation, mucins are hypoglycosylated and these immature forms continuously undergo repeated cycles of internalization, resulting in a more immature form than the mature form. However, on-membrane deglycosylation (enzymatic or chemical) of resolved protein bands did not enhance the reactivity of Mab 2214 with the mature MUC4 bands (data not shown). However, in paraformadehyde fixed cells, MAb 2214 exhibited the highest reactivity with the cell surface. The immature protein is unlikely to be present on the cell surface, and possibly the fixation of cells with paraformaldehyde exposed the MAb 2214 reactive epitope. Further characterization of the low molecular weight form of MUC4 reactive with MAb 2214 is underway.

Immunofluorescence analysis showed specific staining for MUC4 in membranes as well as in the cytoplasmic compartments of HPAF/CD18 cells. The staining pattern was comparable with the anti TR Mab 8G7 and their specificity to MUC4 was further supported by the lack of signal in MUC4 negative cells. The perinuclear staining of Mab 2214 further supports its reactivity to the immature protein.

Due to its large size and multi-domain organization, MUC4 can potentially interact with many proteins and these interactions could be the key to various functions attributed to MUC4. Its interaction with HER2 and the functional significance of this interaction has been well studied [31], [32]. However, there are many other potential interacting partners of MUC4 that could play an important role in modulating or mediating MUC4 function. MAbs 2175 and 2382 were able to immunoprecipitate the MUC4 protein from the cell lysates of HPAF/CD18 cells and could thus help in the isolation and identification of additional MUC4 interacting partners. Further, the predominant reactivity of MAb 2214 to lower molecular weight MUC4 is suggestive of a different form of MUC4 which co-exists with the mature protein. If, in fact, it is the immature form of the protein, then the MAb 2214 may potentially help in the isolation of various novel interacting partners that may interact with this form of MUC4 and unravel its functional significance.

MAbs 2214, 2175 and 2382 also recognized MUC4 expressed in the cancer tissues by immunohistochemical analysis with the reactivity pattern similar to that observed with anti-TR Mab 8G7. None of the normal pancreatic ducts were stained, which is in accordance with our earlier studies that have shown an absence of MUC4 expression in the non-neoplastic ducts. The new antibodies can be useful tools to corroborate the results obtained from 8G7, suggesting the overexpression of MUC4 in various malignancies. Further, due to the non-repetitive nature of their reactive epitope, the newly developed antibodies will provide a more reasonable measure of the extent of overexpression by negating the effects of VNTR polymorphism. The anti-TR antibody 8G7, however, would provide greater sensitivity of detection because of the multiplicity of the epitopes. Thus, the combination of anti-TR and anti-non TR MUC4 antibodies can provide better information about the extent of MUC4 overexpression in the tumor tissues.

Efforts are underway to study the direct inhibitory effects of the antibodies on cancer cell growth, motility and invasion under both *in vitro* and *in vivo* conditions. Our recent studies have demonstrated that MUC4 contributes to the chemoresistance in pancreatic cancer cells by activating anti-apoptotic pathways and promoting cell survival [15]. Hence it will be of interest to study the effect of anti-MUC4 antibodies in inducing apoptosis in cancer cells and augmenting their sensitivity to chemotherapeutic drugs. Further, these antibodies also need to be evaluated for their utility in radioimmunodiagnosis and radioimmunotherapy of MUC4 overexpressing tumors. Functional studies using the non-tandem repeat MAbs may probably provide a better understanding of MUC4 mediated mechanisms in cancer progression. These antibodies could also aid in understanding MUC4 structure-function relationships, regulation of expression and possibly identify a probable interacting partner on the tumor cell surface, which could be the reason for the metastatic phenotype.

In conclusion, our studies indicate that MAbs 2175 and 2382 are highly specific in detecting the non-tandem repeat region of the mucin MUC4 by various immunoassays. These domain specific antibodies would serve as useful reagents to develop quantitative assays, and are valuable tools to study MUC4 structure-function relationships and possibly target MUC4 for therapy of solid tumors that overexpress MUC4.

## Materials and Methods

### Ethics statement

The use of animals for immunization and isolation of spleen was approved by the Institutional Animal Care and Use Committee (IACUC) Protocol # 94-025-12 titled “Monoclonal Antibody Core Facility Immunization Protocol”.

Human pancreatic tumor tissues were obtained from the University of Nebraska Medical Center (UNMC) Tissue Bank and their use was approved via the UNMC Institutional Review Board (IRB) approval # 491-97-EX.

### Generation of recombinant MUC4 domains

Regions of MUC4-α on either side of the TR domain were cloned and expressed, and purified proteins were used as immunogens. Specific primers were designed using MUC4 sequence AJ000281 to amplify the fragments from nucleotides 587 to 3361 [MUC4α-Amino Terminal (MUC4α N-ter)] and from nucleotides 1 to 1293 [MUC4α-carboxy terminal (MUC4α C-ter), representing the regions immediately upstream and downstream of the TR domain, respectively (**Figure 1a**). BamHI and an EcoRI restriction sites were added in the forward and reverse primers, respectively, allowing in-frame cloning with the GST and thrombin cleavage site of the pGEX-2TK vector (Pharmacia). Amplification was done by the expand long RT-PCR system (Roche) as described previously using JER103 and JER109 as templates for sequence AJ00281 and AJ010901, respectively [1]. The constructs were sequenced to confirm the proper reading frame and maintained in E. coli BL21 (New England Biolabs Inc.). A 5 ml overnight preculture of each recombinant strain was used to inoculate 1 liter of 2×YTA medium (16 g tryptone, 10 g yeast extract, and 5 g NaCl in 900 ml of deionized water, 100 µg/ml ampicillin), and grown under agitation at 37C for 3 to 4 h to reach an absorbance at 260 nm between 0.6–0.8, induced by 0.1 mM of IPTG, and cultured for an addition of 3 to 4 h. Cultures were centrifuged and washed three times in ice cold PBS, resuspended in 5 ml of ice cold PBS, and sonicated. Protein lysates were clarified by centrifugation and by filtration on a 0.22 µm filter. Lysates were passed through a 5 ml Glutathione Sepharose Fast Flow column (Pharmacia), washed three times with 5 column volumes of PBS, and eluted with 10 ml of 15 mM reduced gluthatione. Elution fractions of 1 ml were collected and 5 µl aliquot of each fraction was resolved on 10% SDS-PAGE, and proteins detected by coomassie blue staining. Fractions containing pure GST-fusion proteins were pooled and quantified using the BIO-RAD D/C protein estimation kit (BIO-RAD).

### Mouse Immunization

The immunization and selection of MAbs were carried out using established procedures at the UNMC Antibody Core Facility [17]. Briefly, separate groups of mice (BALB/c) were immunized by repeated IP injections of recombinant GST fusion proteins MUC4α-N-Ter and MUC4α-C-Ter at two-week intervals. In each group, immunization with recombinant protein was alternated with the lysate of MUC4 positive HPAF/CD18 human pancreatic cancer cells [17]. Sera from these mice were evaluated in direct binding assays for antibody reactivity with the recombinant MUC4 fusion protein, and GST was used as a negative control. Once an appropriate antibody response was observed in ELISA, the animals were given a final booster injection with the recombinant protein four days prior to exsanguination and splenectomy. Splenocytes were isolated and fused with NS-1 and/or Sp2/0 myeloma cells. Hybridomas producing the antibodies of interest were selected by screening for specific antibody binding to the immunogen of interest (recombinant proteins and HPAF/CD18 lysate) and lack of binding to irrelevant control antigens (GST and BSA).

### Screening for MUC4-positive Hybridomas

Immulon plates were coated with 50 µl of the antigenic preparation (MUC4 recombinant proteins or GST or protein lysates from MUC4 positive cell lines) at a concentration of 2.5 µg/well in bicarbonate buffer (pH 9.6). The plates were incubated overnight at 4°C. The plates were washed in PBST and the free binding sites of the wells were saturated to eliminate non-specific binding of the immunoglobulins by incubating with 200 µl/well of 2% non-fat skimmed milk in PBS for 2 h at 37°C and plates were washed in PBST. One hundred µl of the culture supernatant was transferred from wells of culture plates into corresponding wells in ELISA plates. Mouse pre-immune serum was used as a negative control in each assay, incubated for 1 h at 37°C, and then the plates washed again in PBST. One hundred µl/well of the peroxidase conjugated antibody (anti-mouse HRP, Amersham Biosciences, 1∶2000 dilution in PBS) was added and incubated for 1 h at 37°C. The plates were washed in PBST and 100 µl of TMB substrate (Dako Substrate) was added to each well and incubated at 37°C. The reaction was arrested by adding 100 µl of 2 M sulfuric acid and the plates were scanned at 450 nm in a Biotech ELISA plate reader.

### Immunoprecipitation

Protein lysates from the MUC4-expressing HPAF/CD18 cells were immunoprecipitated using 5 µg/ml of 2382, 2214, 2175, 8G7 (anti-TR antibody), and K2G6 (isotype matched control MAb reacting with KLH). Antigen-Antibody complexes formed were pulled down by using Protein A/G beads (Calbiochem) and the complexes were solublized by using SDS-sample buffer containing 2-mercaptoethanol. The samples were resolved on 2% SDS-agarose gel and were immunoblotted using 8G7.

### Immunoblotting

A series of pancreatic cell lines were processed for protein extraction and Western blotting using standard procedures [17]. Briefly, the cells were washed twice in PBS and scraped in radioimmunoprecipitation assay (RIPA) buffer [50 mM Tris, 5 mM EDTA, 150 mM NaCl, 0.25% sodium deoxycholate; 1% NP40 (pH 7.5)], containing protease inhibitor mixture (Roche Diagnostics, Mannheim, Germany) and phosphatase inhibitors (5 mM NaF and 5 mM Na3VO4; Sigma Chemicals, St. Louis, MO), and kept at 4°C for at least 30 min. Cell lysates were passed through the needle syringe or alternatively subjected to one freeze-thaw cycle to facilitate the disruption of the cell membranes. Cell lysates were centrifuged at 14,000 rpm for 30 min at 4°C, and supernatants were collected. Protein concentrations were determined using a BIO-RAD D/C protein estimation kit. Because of the large size of MUC4, the proteins (20 µg) were resolved by electrophoresis on a 2% SDS-agarose gel under reducing conditions. SDS-PAGE was used for β-actin, (protein loading control), and run under similar conditions. Resolved proteins were transferred onto the polyvinylidene difluoride membrane and subjected to the standard immunodetection procedure using specific antibodies. For MUC4 immunodetection, anti-MUC4 mouse monoclonal antibody 8G7 (1 µg/ml) positive control, and 2 µg/ml of non-tandem repeat antibodies diluted in PBS were used. Anti human β-actin (1∶10000, Sigma AC-15) was used or the protein loading control. Horseradish peroxidase-conjugated goat anti-mouse (Amersham Biosciences) secondary antibody was used at a dilution of 1∶2000. The blots were processed with ECL Chemiluminescence kit (Amersham Biosciences), and the signal was detected by exposing the processed blots to X-ray films (Biomax Films, Kodak, NY).

### Confocal Immunofluorescence Microscopy

For immunofluorescence staining, HPAF/CD18 cells were grown at low density on sterilized glass cover slides overnight. After washing with 0.1 M HEPES containing Hanks buffer, the cells were fixed in ice-cold methanol at −20°C for 2 min. Nonspecific blocking was done in 10% goat serum containing 0.05% Tween 20 for at least 30 min, followed by incubation with the non-TR MAbs 2382, 2214, 2175 and anti-MUC4 TR MAb 8G7 was used as the positive control diluted in PBS. A non-specific isotype matched antibody, K2G6, was used as a negative control (1∶100) for 1 h, at room temperature. Cells were washed 4×5 min with PBS containing 0.05% Tween 20 (PBS-T) and then incubated with FITC conjugated goat anti-mouse secondary antibodies for 30 min. Cells were washed twice with PBS-T, and mounted on glass slides in anti-fade Vectashield mounting medium (Vector Laboratories, Burlingame, CA). Immunostaining was observed under a ZEISS confocal laser-scanning microscope, and representative photographs were captured digitally using 510 LSM software.

### Flow Cytometry

For flow cytometry, cells were harvested non-enzymatically using Cellstripper™ (Mediatech, VA), washed with PBS (1% goat serum) and counted. Cells were fixed for 30 min with 2% paraformaldehyde (in PBS) and blocked with 5% goat serum. Cells were then incubated with indicated antibodies (1 µg/10^6^ cells) for 1 h on ice. Subsequently, cells were washed three times with PBS and incubated with FITC conjugated anti-mouse antibody (0.75 µg/µl, 1∶300 dilution) for 1 h on ice. Cells were washed again three times with PBS and analyzed using the BD FACSCalibur™ flow cytometer.

### Immunohistochemical Analysis

Tissues were fixed in 10% buffered formalin and embedded in paraffin. Sections (5 µm) were cut and processed as described previously. Briefly, tissue sections were deparaffinized in xylene, and rehydrated in graded ethanol. Endogenous peroxidase activity was quenched by incubating sections in 0.3% H_2_O_2_ in PBS for 20 min. Nonspecific binding was blocked by incubating the sections with normal goat-serum for 30 min at room temperature. Sections were then incubated with the anti MUC4 antibody (1∶100) diluted in PBS and a non-specific isotype matched antibody, K2G6, as a negative control for 1 h, at room temperature and washed with PBS-T (3×5 min) followed by incubation with secondary antibody for 30 min. Slides were washed (3×5 min) with PBS-T and incubated with the ABC solution. The reaction color was developed by incubating sections with 3,3′-diaminobenzidine reagent. The slides were washed with water and counterstained with hematoxylin. The sections were then dehydrated in graded alcohols and mounted with Permount permanent mounting media (Fisher Scientific, Fair Lawn, NJ). All slides were observed under Nikon E400 Light Microscope and representative photographs were taken.
